# Reconstruction of extended Petri nets from time series data and its application to signal transduction and to gene regulatory networks

**DOI:** 10.1186/1752-0509-5-113

**Published:** 2011-07-15

**Authors:** Markus Durzinsky, Annegret Wagler, Wolfgang Marwan

**Affiliations:** 1 Magdeburg Centre for Systems Biology, Otto-von-Guericke-Universität, Magdeburg, Germany; 2 LIMOS (Laboratoire d'Informatique, Modélisation et Optimisation des Systèmes), University Blaise Pascal (Faculty of Sciences and Technologies) and CNRS, Clermont-Ferrand, France

## Abstract

**Background:**

Network inference methods reconstruct mathematical models of molecular or genetic networks directly from experimental data sets. We have previously reported a mathematical method which is exclusively data-driven, does not involve any heuristic decisions within the reconstruction process, and deliveres all possible alternative minimal networks in terms of simple place/transition Petri nets that are consistent with a given discrete time series data set.

**Results:**

We fundamentally extended the previously published algorithm to consider catalysis and inhibition of the reactions that occur in the underlying network. The results of the reconstruction algorithm are encoded in the form of an extended Petri net involving control arcs. This allows the consideration of processes involving mass flow and/or regulatory interactions. As a non-trivial test case, the phosphate regulatory network of enterobacteria was reconstructed using *in silico*-generated time-series data sets on wild-type and *in silico *mutants.

**Conclusions:**

The new exact algorithm reconstructs extended Petri nets from time series data sets by finding all alternative minimal networks that are consistent with the data. It suggested alternative molecular mechanisms for certain reactions in the network. The algorithm is useful to combine data from wild-type and mutant cells and may potentially integrate physiological, biochemical, pharmacological, and genetic data in the form of a single model.

## Background

Network reconstruction methods infere mathematical models of real world networks directly from experimental data ([1-5] and references therein). We have recently described an approach to the reconstruction of causal interaction networks from time series data sets [6,7]. The original algorithm has two significant properties. (1) It delivers provenly ALL minimal networks which are able to reproduce the time series data that served as input and (2) the algorithm is exact as it does not involve any heuristic decisions by the operator so that the results are independent of any personal bias. Having a complete list of alternative networks which are compatible with experimental data shall facilitate the design of new experiments aimed at ruling out alternatives to systematically find a final, unique solution.

**Figure 1:**
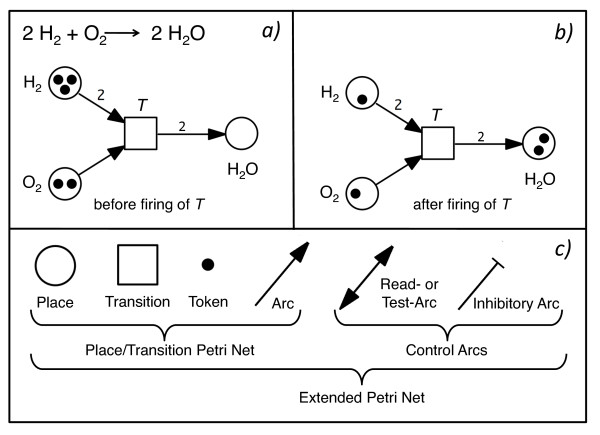
**Petri net elements and the representation of a chemical reaction in the form of a Petri net**. a) Petri nets are weighted, directed, bipartite graphs consisting of nodes and arcs. The nodes of a Petri net, places and transitions, are interconnected by arcs. An arc always connects a place with a transition or *vice versa*, but never two places or two transitions with each other. Places may contain (be marked with) tokens, while transitions move tokens rather than containing them. Petri nets provide a useful, mechanism-oriented framework to represent chemical or biochemical reactions. Panel (a) shows a Petri net model of the synthesis of water from its elements, hydrogen and oxygen. Individual molecules are represented by tokens in the respective places. In the example shown, two out of three molecules of hydrogen react with one molecule of oxygen to give two molecules of water. The chemical reaction is modeled by firing of the transition *T*. Numbers adjacent to arcs are called arc weights. When no number is given, the arc weight by definition is one. The arc weight indicates how many tokens are removed from a place (outgoing arc) or moved into a place (ingoing arc) when a transition fires. Arc weights can be used to represent the stoichiometry of a chemical reaction, accordingly. After two molecules of water have been formed (b), the transition cannot fire a second time because one molecule of hydrogen is missing so that the stoichiometry of the reaction is not fulfilled. c) Standard Petri nets, so-called place/transition nets are composed of places, transitions, tokens and (standard) arcs. Extended Petri nets in addition have read arcs and/or inhibitory arcs that put an additional constraint on the firing licence of a transition, depending on the marking of its pre-places (for details see Figure 5).

**Figure 2:**
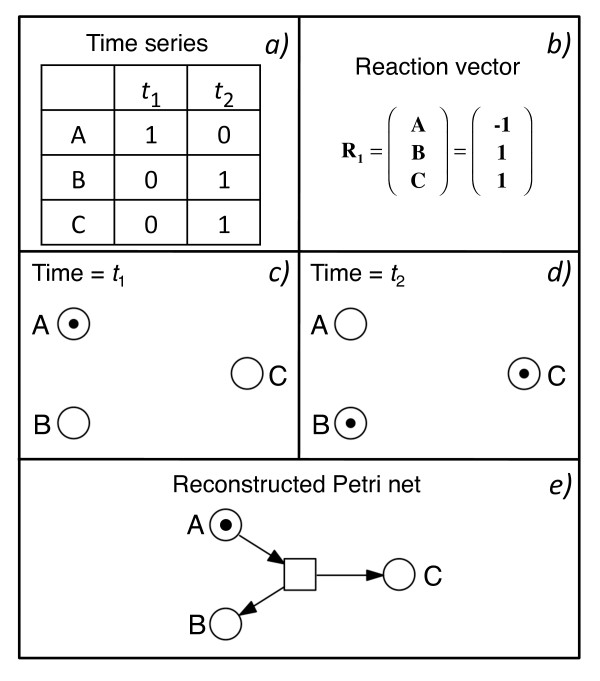
**The principle of automatic network reconstruction explained with the help of a trivial example**. a) The input for the reconstruction algorithm is a time series data set that describes the time-course of the components of interest (A,B,C) with discrete values as a causal sequence of events. At time *t*_2 _the system reached its terminal state, i.e. the values of all components have reached their final level. In the simplest form, the entries are boolean (0,1). b) Shows the reaction vector of the transition in e). A reaction vector corresponds to the incidence matrix of an individual transition or to a column in the incidence matrix of a Petri net. c,d) The presence of the components at given time points is represented by tokens in places assigned to the components. The algorithm evaluates those places the marking of which has changed between two successive time points and e) connects these places with transitions that cause the observed flow of tokens in the reconstructed Petri net.

The output of the algorithm can be encoded as simple place/transition Petri net (Figure 1; [8]) containing only the minimal number of nodes and arcs required to fit the given data set. In order to exactly reproduce the experimental observations, we additionally use priorities among transitions to enforce an order in which competing transitions fire [6]. The priorities reflect relative kinetic rate constants. The algorithm starts by assigning one place to each (biochemical) component or factor which has been measured in the form of a time series and tries to connect these places by a minimal set of transitions (Figure 2). Transitions may be interpreted as (bio-) chemical reactions ([9-11] and references therein). If the number of components measured in the time series is not sufficiently high in order to create a Petri net which is able to reproduce the data, the algorithm adds one place and restarts the reconstruction process and continues to do so until solutions are found [6,7].

For the trivial example shown in Figure 2 the solution of the reconstruction problem is obvious: The two successive states of the system which are reflected by the time series data set (Figure 2a) are the result of the firing of a single transition connecting three places (Figure 2e). In more complex data sets however, the differences measured between two successive time points may have been caused by the firing of more than one transition. Therefore the algorithm has to identify all combinations of putative transition firing events the sum of which might lead to the difference observed between two successively measured time points (see [6] for details).

**Figure 3:**
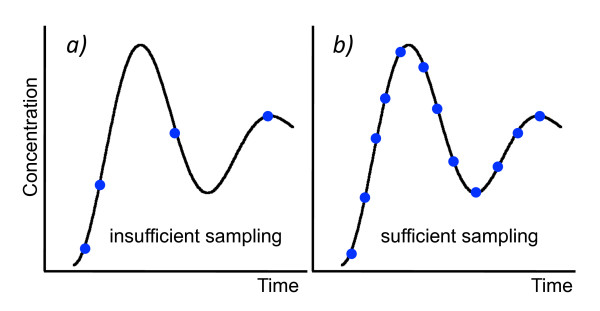
**Constraints for the quality of data to be suitable for automatic network reconstruction**. The algorithm requires that the time course of the considered components is known, no matter whether the data are boolean or continuous (and are subsequently discretized) as only those changes are considered that in fact are reflected by the data set. a) The number of time points measured is so low that the time course of the signal is not reflected with a sufficient resolution in detail and interpolation of the data points may mislead the algorithm as phases of formation and decay of the component are missed. b) The number of time points is sufficiently high in order to correctly reflect the time course of the component.

**Figure 4:**
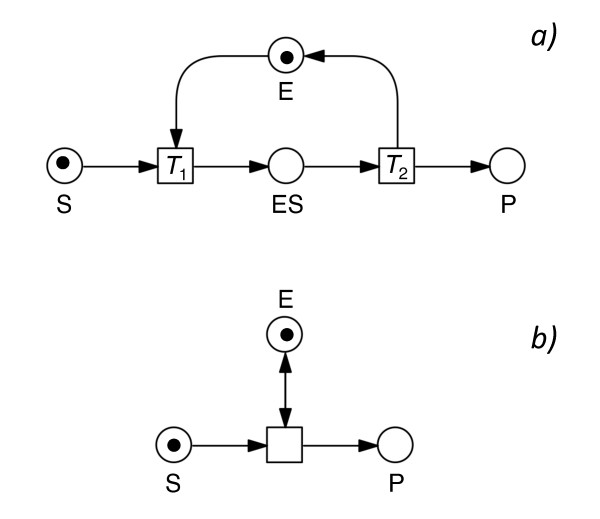
**Alternative ways to represent a catalytic (enzymatic) reaction**. a) An enzymatic reaction converting substrate S into product P is represented in the form of a place/transition Petri net by modeling the formation and decay of the enzyme-substrate complex ES. Upon firing of *T*1, enzyme E is consumed and released upon firing of *T*2. b) In the framework of an Extended Petri net, an enzymatic reaction can be modeled with a test arc (bidirected arc) connecting the Enzyme place E with the transition that converts substrate S into product P. The transition can only fire if there is at least one token in E. Upon firing of the transition, the token is consumed from and immediately redelivered into place E, i.e. unlike in a), the marking of E does *not *change while the token of place S moves to place P.

According to the sampling theorem, the number of time points taken in a series needs to be sufficiently high to correctly capture the time-dependent change of the measured components in the form of a time-discrete characteristics (Figure 3). Potential oscillations of individual components which occur asynchronously or which are by far too fast to be observed at the time scale of interest (e.g. formation and decay events of individual enzyme substrate complexes; Figure 4a) were not considered by the algorithm as this would cause an explosion of solutions [12] and because such events cannot be observed anyway. It follows that the original reconstruction algorithm [6,7] considers only macroscopic changes that are measured within the chosen time scale of a given data set. The resulting assumption of monotonicity of the time course of the components in turn puts a severe limit to the formal representation of any form of catalysis or inhibition if, as in [6], only standard Petri nets (*i.e*. Petri nets without contol arcs) are used for the reconstruction process. As the formation and the decay of enzyme-substrate complexes in most cases are so fast and do occur asynchronously at the level of individual molecules, individual turnovers (Figure 4a) are usually not resolved by experimental time series measurements. Representing the formation and decay of the enzyme-substrate-complex of an enzymatic (catalytic) reaction by a single transition that directly connects the two places substrate (S) and product (P) of the enzymatic reaction through a bidirected arc (read arc; Figure 4b) solves this dilemma. In fact, our originally described algorithm does not work with so-called extended Petri nets which in addition to standard arcs may contain read arcs and inhibitory arcs. In order to include catalytic and inhibitory reactions, we had to fundamentally redesign our network reconstruction algorithm. Considering catalysis and inhibition significantly extends the practical applicability over the original algorithm [6] as demonstrated in the present paper. The new algorithm now allows for the reconstruction of signal transduction and gene regulatory networks. It also allows the use of data sets that compare the behaviours of wild-type and mutant cells.

Although the concept of providing a complete list of solutions remains and the basic principle of reconstructing the network in part is the same as in reference [6], this work is based on fundamental methodological improvements [13] (Durzinsky, M., W. Marwan, A. Wagler: Reconstructing extended Petri nets, submitted) of the underlying mathematics as compared to refs. [6,7]:

(1) While in [6] all possible reaction sequences are enumerated by simulation, the new approach uses an implicite representation of all possible combinations, thereby avoiding combinatorial explosion.

(2) Terminal states of the network are now applied in a new way which again reduces the number of combinations to be analysed.

(3) Reaction vectors and catalytic events in the form of control arcs are now treated completely independent from each other by introducing control functions. This again greatly reduces the number of combinations to be analysed.

(4) The new algorithm allows that reactions may depend on arbitrary logic combinations of the presence or absence of components allowing to represent complex regulatory dependencies.

A detailed description of the mathematical model extended by the new concept of control functions and the mathematical proof of the completeness of solutions provided by the algorithm will be publised in a compagnion paper [13] (Durzinsky, M., W. Marwan, A. Wagler: Reconstructing extended Petri nets, submitted). In this manuscript, we describe its application to the reconstruction of signal transduction and gene regulatory networks.

A standard experimental approach in molecular biology is to introduce structural changes into a network and to study the resulting change in its static or dynamic behaviour. Such structural changes can be introduced by genetic or pharmacological intervention, for example. Many reactions in a biochemical network depend on the presence or the absence of a certain component while there may be no obvious, measurable time-dependent change in concentration or abundance of these respective components. Enzymes catalyze biochemical reactions, like e.g. the phosphorylation or dephosphorylation of proteins in a regulatory network. Formally, and based on the original definition of catalysis by Berzelius, a gene may be seen to catalyse an even far downstream process. Deleting an enzyme-encoding gene for example may abolish a certain biochemical reaction. Although transcription and translation are in between, the gene indirectly acts as a catalyst of the biochemical reaction in the sense that the gene is necessary for the biochemical reaction to occur, but it is not consumed by the reaction.

**Figure 5:**
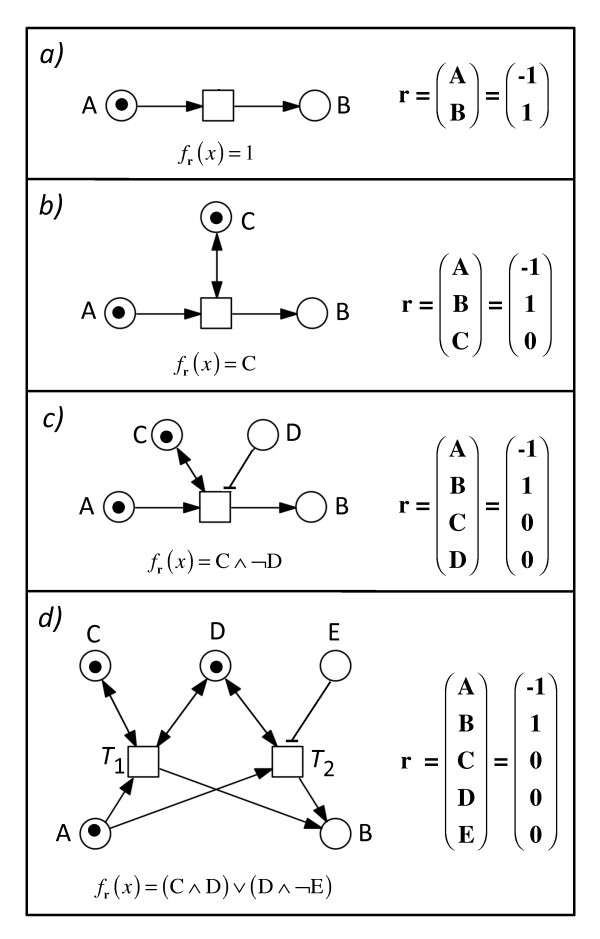
**Implicit representation of extended Petri nets by controled reactions**. A controled reaction ***R_c _*= (r,*f*_r_) **is composed of the reaction vector **r **and the associated control function ***f*_r_**. The arcs of an extended Petri net can be thought of to consist of two sets. (1) The standard arcs and (2) the control arcs (bidirected arcs and inhibitory arcs). A reaction vector describes how the marking of the connected places changes upon firing of a transition. The control function defines the conditions under which firing of at least one of all transitions with the same reaction vector may occur. The marking of the places of all four Petri nets shown in panels a) to d) has been choosen such that all transitions can fire. If there is a token in place A, the transition in a) can always fire, the transition in b) can only fire if there is at least one token in place C, while the transition in c) can only fire if there is at least one token in place C and if place D is empty. In the Petri net of panel d), the token has two options to move from place A to place B. It can move through firing of *T*_1 _if there is at least one token in place C and at least one token in place D. The alternative path, firing of *T*_2_, requires that there is at least one token in place D while place E must be empty. In panel d), the reaction vector of the controled reaction ***R_c _*= (r,*f*_r_) **represents the set of two transitions *T*_1 _and *T*_2 _each of which connects the places A and B in the same direction through standard arcs. Note that if places A in panels a) to d) do not contain any token, none of the transitions could fire. Symbols: ∧, logic AND; ∨, logic OR; -, logic NOT.

Reactions in biochemical or genetic networks may be subject to the control by inhibitors as well. Deletion of a gene encoding an inhibitory subunit of a specific protein may render this protein constitutively active. A biochemical reaction or the expression of a gene may be controled by different factors and/or might occur through alternative mechanisms. The described regulatory dependencies of reactions can be modeled with the help of read arcs (bidirected arcs) and inhibitory arcs in the extended Petri net formalism. These control arcs determine whether a given transition is able to fire depening on the marking of its pre-places (Figure 5). The reader interested in the mathematical details of the algorithm and the proof of its correctness is referred to the compagnion paper [13].

## Results

Neglecting catalysis and inhibition, each transition *T *can be encoded by a reaction vector **r**^*T *^and the collection of all such vectors yields the incidence matrix of the studied network. Considering catalysis and inhibition we deal with controled reactions: Each set of such transitions that connect the same places in the same way is encoded by a controled reaction ***R_c _*= (r, *f*_r_)**, a pair, where is the reaction vector indicating the change in the marking of places caused by firing of any of the transitions of the set and ***f*_r _**is a control function encoding control arcs connected to the transitions. In other words, reaction vectors and control functions separately describe two different structural properties of one and the same extended Petri net: (1) The reaction vectors **r **describe how the places of a Petri net are connected by transitions through directed standard arcs, and how the marking of the places accordingly changes upon firing of the transitions. (2) The control function ***f***_**r **_describes which places are connected to given transitions through control arcs (read arcs or inhibitory arcs, respectively; Figure 5). The control function also defines the conditions under which firing of at least one of all transitions with the same reaction vector can occur. The control function is 1 (TRUE) for a given transition if the marking of all pre-places that are connected by control arcs to that transition licenses this transition to fire. When a transition is not connected to any control arc, the value of the control function ***f***_**r **_is one and the firing of the transition depends only on the marking of its pre-places (Figure 5a). When a transition is connected by a control arc (read arc or inhibitory arc) to a place, the control function is 1 and the transition is allowed to fire only if the marking of this place is as defined by the control arc. If the control arc is a read arc as in the example of Figure 5b, the condition for firing is fulfilled, only if there is at least one token in the controling place (in place C in Figure 5b). If the control arc is an inhibitory arc, the condition is fulfilled only if there is no token in the pre-place connected by the inhibitory arc to the transition. If the firing of a given transition is under the control of more than one control arc (read arc or inhibitory arc), the control function is one if all conditions are fullfilled. In the Petri net of Figure 5c this is the case if there is at least one token in C and if there is no token in D. A controled reaction **r **may eventually represent more than one transition of the same reaction vector. (This is why we discriminate between reaction vectors **r **and transitions *T *by using different symbols throughout.) In this case the control function represents all conditions for the firing of any of the transitions of this set, indicating whether the reaction can occur by firing of any one of those transitions (Figure 5d). The marking of the regulating places may indeed be updated through transitions connected through standard arcs to other places of the network.

### Essential function of the algorithm

**Figure 6:**
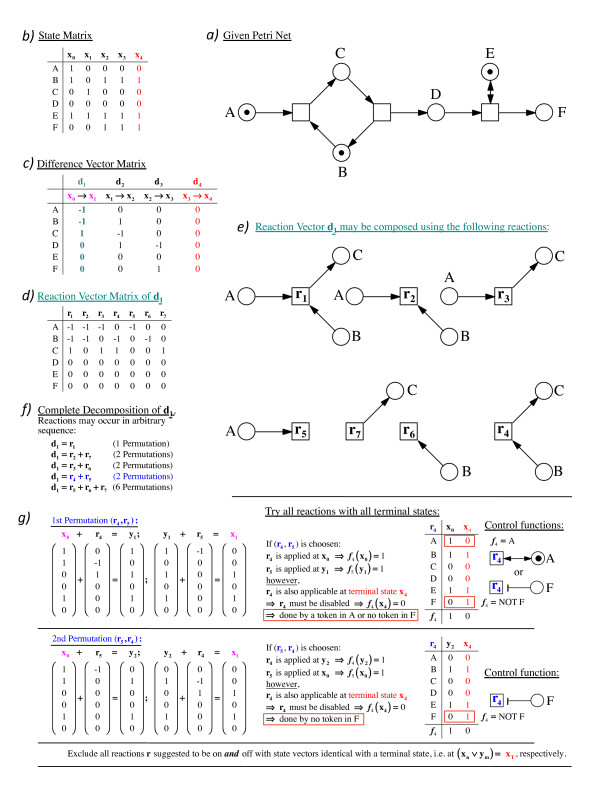
**Essential steps in the reconstruction of an extended Petri net**. The simple extended Petri net with places A to F as shown in panel a) was used to generate a time series data set which in turn was taken to explain essential the steps of network reconstruction as shown in panels b) to g).

We describe the essential function of the algorithm by taking the small Petri net of Figure 6a as an example. The small net is used to generate a discrete time series data set starting with the initial marking shown. In this context, a time series data set indicates how the values of the components (here represented as the marking of the places) change as a function of time. As the marking of the net may not necessarily change with each measured time point, the time series data set is compiled into a state matrix (Figure 6b) which indicates how the marking of the net develops as the tokens pass through until a terminal state is reached (**x_4 _**in Figure 6b). A terminal state is defined as a state where the marking of all places in the Petri net does not change anymore. Compilation eliminates redundancy in the time series data by deleting information for time points where no change was detected as compared to the previous measurement. Information from several time series data sets obtained in different experiments and/or performed with networks after structural intervention (e.g. with cells that carry mutations in the pathways of interest) may be combined into a single state matrix to be used as input for the reconstruction algorithm (see case study; not shown in Figure 6).

From the state matrix, the difference vector matrix is computed with difference vectors indicating how the marking of the net (i.e. the number of tokens in all places) changes from one state to the next. The two states **x_0 _**and **x_1_**, the difference vector **d_1 _= x_1 _- x_0_**, and the terminal state **x_4 _**are arbitrarily chosen to demonstrate subsequent steps of the algorithm. As it is not known *a priori *whether the change in marking of places represented by a given difference vector is caused by the firing of one or of several transitions, all difference vectors are decomposed into the maximal sets of possible individual reaction vectors **r **to be listed in one reaction vector matrix. The reaction vector matrix lists all possible connections of places and transitions by standard arcs as putative nodes of a reconstructed Petri net. Which of these connections indeed is suitable to be a transition in alternative reconstructed networks and in which temporal order the transitions fire is estimated at a later stage of the analysis. Figure 6d shows the decomposition of the difference vector **d**_1 _= (-1, -1, 1, 0, 0, 0)^*T *^into seven different reaction vectors as an example. For the sake of clarity, the reaction vectors obtained by exhaustive decomposition of **d_1 _**are graphically represented as Petri nets in Figure 6e. Referring to a biochemical model, complete decomposition takes into account that it is not known whether the chemical components A, B, and C react with each other or (in part) separately. In case A, B and C are involved in more than one chemical reaction, it is open in which temporal sequence these reactions do occur. Therefore, all possible permutations of all reaction vector combinations that give a difference vector have to be considered (Figure 6f). This may however lead to intermediate states of the system that have not been observed in the time series data set. This is demonstrated by taking the two possible permutations of the sequence of **r_4 _**and **r_5 _**as example (Figure 6g). In the first permutation (the 1st reaction is **r_4 _**and the 2nd reaction is **r_5_**), the system proceeds from state **x_0 _**to state **x_1 _**through an additional, intermediate state **y_1_**. In the second permutation (the 1st reaction is **r_5 _**and the 2nd reaction is **r_4_**), the additional, intermediate state is **y_2_**. For the sake of completeness it is essential to compute all potential additional, intermediate states and this complete set is required for performing the next step of the algorithm in which all reactions that cannot be part of a functional reconstructed Petri net are filtered out. Filtering is performed by testing all reactions of the reaction vector matrix with all states of the state matrix which has been extended by all additional states **y**. All sums of a state vector and a reaction vector are evaluated. This is performed by asking which reaction is applicable in each individual state of the system (as listed in the state matrix). A reaction, by definition, is applicable to a state if it does not generate an invalid successor state with entires out of bound (corresponding to a negative or a too large number of tokens per place). The remaining pairs of state vector and applicable reaction vector are analysed for the potential control by the control function the value of which may change in a marking-dependent manner while the tokens flow through the net.

Each individual transition might potentially be under the control of test- and/or of inhibitory arcs that might emerge from ANY of the places and hence all reactions must be considered for such potential control. If a reaction is applied to (occurs at) a given state, the control function ***f*_*r *_**must have the value of 1 at that state. If a reaction is applicable to one of the terminal states (to terminal state **x_4 _**in the example of Figure 6b), the reaction must be disabled at this state, because otherwise the state would not be terminal. In this case the control function must be ***f*_*r*_(*x*_4_)= 0**. By comparison of state vectors where the reaction is enabled and state vectors of terminal states where the reaction has to be disabled, one then identifies potential places, the marking of which might control the firing licence of the respective transition of a Petri net in terms of an argument of the control function. In the example shown in Figure 6g (right side, 1st permutation), reaction **r_4 _**occurs at the state **x**_0 _which has a token in A but no token in place F. Because the terminal state **x**_4 _(at which **r**_4 _must be disabled) has no token in A but a token in F, either of the two places can control **r**_4_. In other words, the reaction **r**_4 _may be mediated by a transition which is connected to place A by a test arc, or it may be mediated by a transition which is connected to place F by an inhibitory arc. Note that, in the simple case, only one of the two controled reactions (**r**_4_, *f*_4 _= A) or (**r**_4_, *f*_4 _= -F) needs to be chosen to compose the reconstructed Petri net. When a reaction occurs at multiple different states and has to be disabled at multiple terminal states, more complex control is possible (Figure 5).

To summarize, when a reaction **r **is applied to a certain state **x**, it has to be enabled by its control function at that state **(*f*_r_(x) = 1)**. If the reaction is applicable at some terminal state **x'**, the control function has to disable the reaction **(*f*_r_(x') = 0)**, otherwise **r **could produce a successor state of **x' **contradicting its property to be a terminal state. With respect to their associated control function ***f*_r_**, reactions **r **as elements of a controled reaction ***R_c _*= (r, *f*_r_) **fall into three groups:

(I) A reaction that is not applicable to any terminal state can be enabled at all times ***f***_**r **_**≡ 1**) which means that by minimality no control arcs are pointing to the respective transition in the reconstructed Petri nets.

(II) A reaction which is applicable to an intermediate state of the system which has the same state vector **x **as a terminal state has, must be deleted (ruled out), because the control function ***f***_**r **_cannot be 1 (on) and 0 (off) at identical marking of the Petri net. Hence no appropriate control arcs exist in this case.

(III) In all other cases the control of the reaction by control arcs is possible (Figure 6g; right side).

This allows to identify places that potentially do control the reaction through a control arc. Mathematical details are given in [13].

**Figure 7:**
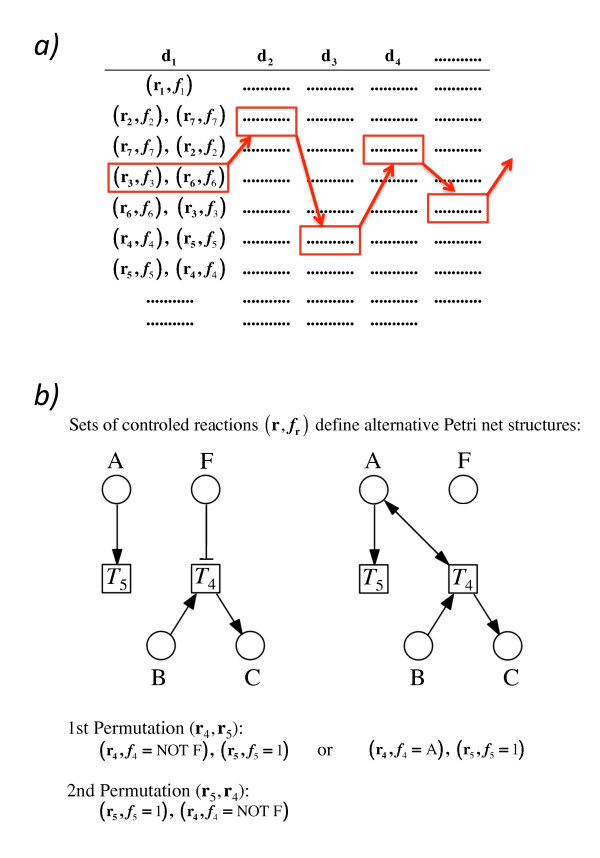
**Composition of Petri nets from controled reactions**. a) The algorithm described with the help of Figure 6 provides the complete set of possible controled reactions ***R_c _*= (r,*f*_r_) **for each difference vector **d_m_**, here arrayed in a table where all possible controled reactions of subsequent difference vectors are listed in subsequent columns. Any arbitrary sequence of controled reactions obtained by taking one difference vector from each of the subsequent columns gives one functional extended Petri net which is compatible with the time series data set that originally served as input [7]. There are no combinations of controled reactions generated in the described way that would give dysfunctional Petri nets because all invalid or contradictory reaction vectors have been filtered out as described. Red arrows indicate one possible trajectory of assembling a valid Petri net. Panel b) shows two reconstructed network motifs according to sets of controled reactions computed by the algorithm for difference vector **d_1 _**(see Figure 6) both of which are compatible with the input data: in a molecular interpretation, chemical reaction of B to C may depend on the absence of F (left) or the presence of A (right). A vanishes in a separate reaction in both cases. The two network motifs shown correspond to the decomposition **d**_1 _= **r**_4 _+ **r**_5 _(see Figure 6g). None of the two displays the wiring of the original Petri net (Figure 6a) which can be retrieved using the decomposition **d**_1 _= **r**_1_.

Exhaustive decomposition of all difference vectors into reaction vectors with all possible permutations in the order (sequence) of the reactions and testing them against the state vectors finally yields a complete list of controled reactions **(*R_c _*= (r,*f*_r_)) **as potential elements of a reconstructed extended Petri net. Reverse engineered Petri nets are then composed simply by combining for each difference vector one arbitrarily chosen set of controled reactions that results from the decomposition of each of the subsequent difference vectors. Figure 7a shows one possible trajectory for obtaining one possible Petri net that is consistent with the input data set. All Petri nets obtained accordingly through different trajectories are compatible with the input data [13]. An example of how sets of controled reactions translate into Petri net structures is shown in Figure 7b. The lower the number of sets of controled reactions found for any given difference vector is, the lower is the number of alternative Petri nets that are compatible with the input data set. A unique Petri net is obtained if there is only one reaction set for each difference vector. If more or even many alternative Petri nets are obtained, the implicit representation of solutions is useful for the design of experiments as it directly shows which parts of the underlying network are still ill-defined.

### The phosphate regulatory network as a test case

The phosphate regulatory network is a network of interacting phosphate-sensing and signal transducing proteins regulating the expression of a battery of genes which are arranged in the *pho *operon. These jointly controlled genes encode proteins related to the phosphate metabolism of enteric bacteria [14,15]. The network serves as a non-trivial test case as the corresponding Petri net contains negative feed-back loops [16] which have to be recognized by the reconstruction algorithm. In the following, we will first briefly describe the biological function of the phosphate regulatory network and its implementation in the form of a Petri net model as far as it is relevant for understanding the *in silico *experiments on genetic (structural) perturbation. Subsequently, we will describe the *in silico *experiments performed to obtain the time series data set. Finally, we will demonstrate step by step how the network was reconstructed on the basis of this data set.

#### Biology of phosphate regulation

**Figure 8:**
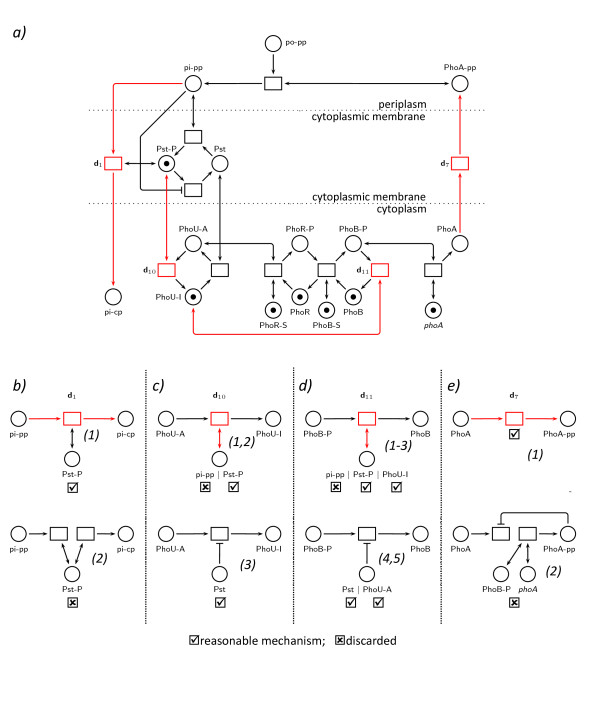
**Implicit representation of the phosphate regulatory network reconstructed and graphically displayed in extended Petri net format**. a) This Petri net model of the phosphate regulatory network in enteric bacteria [16] was predefined and used to perform the *in silico *experiments listed in Table 1 to give the data set compiled into the state vectors of Tables 3 and 4. Phosphorylation sites of the PhoR and PhoB proteins are represented as places (PhoR-S and PhoB-S, respectively). Site-directed deletion of the phosphorylation sites is modeled by removing the token from the respective places to give the initial marking of the Petri net used for simulation of the token flow in the mutants. The net shown in a) was also returned as one result of the reconstruction algorithm. It represents the combination of one controled reaction choosen for each difference vector obtained as shown in Figure 7a. The core of the Petri net as drawn in black is identical in all solutions. However, there are alternatives to the wiring of transitions and places due to the existence of more than one controled reaction for each of the corresponding difference vectors. Transitions with alternative wiring are drawn in red. The alternative controled reactions for each of those difference vectors are shown in panels b) to e). A detailed explanation of these alternatives is given as supplementary material in Additional file 1.

Growth of micro-organisms requires the presence of inorganic phosphate (P_i_), an essential component for the synthesis of nucleic acids (DNA and RNA). Inorganic phosphate is taken up by the PstSCAB complex, which transports inorganic phosphate into the cytoplasm against its concentration gradient (see legend of Figure 8 for details). Under natural growth conditions, the concentration of inorganic phosphate may become a growth-limiting factor. When inorganic phosphate is absent, the phosphate transporter lacks substrate and relays this information through a cascade of three proteins, PhoU, PhoR, and PhoB to cause, among others, the transcription of the *phoA *gene which encodes alkaline phosphatase. This enzyme is synthesized and exported from the cytoplasm into the periplasm where it accumulates in considerable amounts. By degrading organic phosphate compounds that may originate from decaying organisms, alkaline phosphatase provides inorganic phosphate which is taken up into the cell by the PstSCAB complex. As the biosynthesis of high amounts of alkaline phosphatase is energetically expensive, transcription of the *phoA *gene is tightly controled by the concentration of inorganic phosphate through negative feed-back loops that stop the transcriptional activation of the *phoA *gene when sufficient Pi is available.

Note that this paper does not make any scientific contribution to the biology of phosphate regulation. The Petri net model of the phosphate regulatory network is only used as a test case for the network reconstruction algorithm.

#### Petri net model of the phosphate regulatory network

The biochemical interaction of the proteins of the phosphate regulatory network and established feed-back mechanisms [15] have been translated into a Petri net model (Figure 8) [16] with the help of the Petri net tool Snoopy [9,17,18]. Influences of protein conformation on the activity of biochemical reactions, e.g. the phosphorylation of PhoR caused by the active form of PhoU has been modeled by read arcs. Using the simulation mode of Snoopy, the response of the network to addition and removal of inorganic phosphate has been analysed *in silico *to obtain time series data sets. *In silico *experiments were chosen to mimic common experimental methodology and, therefore, produce realistic input data for the reconstruction.

#### Reconstructing the phosphate regulatory network

For reconstructing the phosphate regulatory network, we started with protein components. The task was to find the wiring diagram based on the simulated time-series data sets. The time series data indicated the phosphorylation status of PstSCAB, PhoR, and PhoB in response to external inorganic phosphate. We did not use any kinetic information on the interconversion of the PhoU protein between its inactive and its active state supposing that the corresponding conformational change cannot be directly measured, but the algorithm was told that these two forms, active and inactive, exist. We then generated *in silico *deletion mutants for the *pstSCAB, phoU, phoR*, and *phoB *genes by adjusting the initial marking of the net accordingly (Tables 1,2) and analysed the response of each of those mutants to the removal of inorganic phosphate as compared to the wild-type.

**Table 1:** *In silico *experiments performed to obtain time series data sets on wild-type cells and deletion mutants by following the token flow through the Petri net of Figure 8a

Experiment	Genetic background	Experimental perturbation	Petri net implementation
Exp #1	Wild-type	Addition of organic and inorganic phosphate	token in pi_pp and po_pp
Exp #2	Wild-type	Absence of organic and inorganic phosphate	Petri net as shown in Figure 8
Exp #3	Wild-type	Inhibition of transcription/translation	no token in *phoA*, token in po_pp
Exp #4	Δ *pstSCAB*	Absence of organic and inorganic phosphate	no token in Pst-P
Exp #5	Δ *pstSCAB*	Addition of organic phosphate	no token in Pst-P, token in po_pp
Exp #6	Δ *pstSCAB*	Addition of organic and inorganic phosphate	no token in Pst-P, token in pi_pp and po_pp
Exp #7	Δ *phoU*	Absence of organic and inorganic phosphate	no token in PhoU-I
Exp #8	Δ *phoR*	Absence of organic and inorganic phosphate	no token in PhoR
Exp #9	Δ *phoB*	Absence of organic and inorganic phosphate	no token in PhoB
Exp #10	*phoR *Δ Psite	Absence of organic and inorganic phosphate	no token in PhoR-S
Exp #11	*phoB *Δ Psite	Absence of organic and inorganic phosphate	no token in PhoB-S

For each experiment, the type of experimental perturbation and the implementation of this perturbation in the form of the intitial marking of the Petri net of Figure 8a is indicated. Genetic background column: Δ*phoR *means that the *phoR *gene is deleted and that the PhoR protein is absent accordingly, *etc*. ΔPsite means that the phosphorylation site in the respective protein is deleted, while the protein as such is present.

**Table 2:** Modeled molecular events that, starting from the initial marking, lead to successive states of the Petri net

Process	leads to State
Initial marking, inorganic phosphate present	x0
Uptake of Pi, depleting Pi from the periplasm	x1
Dephosphorylation of PstSCAB-P	x2
Activation of PhoU	x3
Phosphorylation of PhoR	x4
Phosphorylation of PhoB	x5
Biosynthesis of PhoA	x6
Transport of PhoA into the periplasm	x7
Degradation of Po_PP (organic phosphate)	x8
Phosphorylation of PstSCAB	x9
Deactivation of PhoU	x10
Dephosphorylation of PhoB-P	x11

The table is useful to assign the state vectors listed in Tables 3 and 4 to the subsequently occurring molecular events.

Starting with the initial marking as shown in Figure 8a, we generated a time series data set for the wild-type by allowing the tokens to step by step move through the net, which directly gave the first part of the state matrix (Tables 3 and 4; see also Figure 6). The state matrix was subsequently extended by adding compiled time series data for all experiments listed in Table 1 to represent the subsequent occurrence of all states of the system observed in all *in silico *experiments in the form of one comprehensive data set. The state matrix was then used to calculate the difference vector matrix as described (see Figure 6). The difference vectors obtained in all experiments are listed only once, i.e. the same difference vector observed in several experiments gives only one entry as a column in the difference vector matrix. Decomposition of the difference vectors into all possible sums of reaction vectors yielded the reaction vector matrix. At this point P-invariants restricted the number of possible reactions.

**Table 3:** State vectors used for reconstructing the phosphate regulatory network

Exp. #	1												2									3						4	5	6	7		8		
	State vectors (compiled from time-series)																																		
Vector #	0	1	2	3	4	5	6	7	8	9	10	11	12	13	14	15	16	17	18	19	20	21	22	23	24	25	26	27	28	29	30	31	32	33	34
**pi-pp**	1	0	0	0	0	0	0	0	1	1	1	1	0	0	0	0	0	0	0	0	0	0	0	0	0	0	0	0	0	1	0	0	0	0	0
**pi-cp**	0	1	1	1	1	1	1	1	1	1	1	1	0	0	0	0	0	0	0	0	0	0	0	0	0	0	0	0	0	0	0	0	0	0	0
**po-pp**	1	1	1	1	1	1	1	1	0	0	0	0	0	0	0	0	0	0	0	0	0	1	1	1	1	1	1	0	1	1	0	0	0	0	0
**Pst-P**	1	1	0	0	0	0	0	0	0	1	1	1	1	0	0	0	0	0	0	0	0	1	0	0	0	0	0	0	0	0	1	0	1	0	0
**Pst**	0	0	1	1	1	1	1	1	1	0	0	0	0	1	1	1	1	1	1	1	1	0	1	1	1	1	1	0	0	0	0	1	0	1	1
**PhoU-I**	1	1	1	0	0	0	0	0	0	0	1	1	1	1	0	0	0	0	0	0	0	1	1	0	0	0	0	1	1	1	0	0	1	1	0
**PhoU-A**	0	0	0	1	1	1	1	1	1	1	0	0	0	0	1	1	1	1	1	1	1	0	0	1	1	1	1	0	0	0	0	0	0	0	1
**PhoR**	1	1	1	1	0	1	1	1	1	1	1	1	1	1	1	0	1	1	1	1	0	1	1	1	0	1	0	1	1	1	1	1	0	0	0
**PhoR-P**	0	0	0	0	1	0	0	0	0	0	0	0	0	0	0	1	0	0	0	0	1	0	0	0	1	0	1	0	0	0	0	0	0	0	0
**PhoR-S**	1	1	1	1	1	1	1	1	1	1	1	1	1	1	1	1	1	1	1	1	1	1	1	1	1	1	1	1	1	1	1	1	1	1	1
**PhoB**	1	1	1	1	1	0	0	0	0	0	0	1	1	1	1	1	0	0	0	0	0	1	1	1	1	0	0	1	1	1	1	1	1	1	1
**PhoB-P**	0	0	0	0	0	1	1	1	1	1	1	0	0	0	0	0	1	1	1	1	1	0	0	0	0	1	1	0	0	0	0	0	0	0	0
**PhoB-S**	1	1	1	1	1	1	1	1	1	1	1	1	1	1	1	1	1	1	1	1	1	1	1	1	1	1	1	1	1	1	1	1	1	1	1
**PhoA-T**	1	1	1	1	1	1	1	1	1	1	1	1	1	1	1	1	1	1	1	1	1	0	0	0	0	0	0	1	1	1	1	1	1	1	1
**PhoA**	0	0	0	0	0	0	1	0	0	0	0	0	0	0	0	0	0	1	0	1	1	0	0	0	0	0	0	0	0	0	0	0	0	0	0
**PhoA-pp**	0	0	0	0	0	0	0	1	1	1	1	1	0	0	0	0	0	0	1	1	1	0	0	0	0	0	0	0	0	0	0	0	0	0	0

**Term. state**																					T						T	T	T	T		T			T

States are assigned to the experiments during which they occurred. The state vectors of experiments 1 to 8 are shown in this table, the state vectors of experiments 9 to 11 are shown in Table 4. States of Pst, PhoU, PhoR, and PhoB were considered as P-invariants. Phosphate, PhoR, PhoRP and the cytoplasmic PhoA protein were excluded as catalytic factors based on biological knowledge.

**Table 4:** State vectors used for reconstructing the phosphate regulatory network, difference vectors and additional decomposed reactions as obtained in the course of network reconstruction

Exp. #	9				10			11																						
	State vectors (compiled from time-series)											Difference vectors											Additional decomposed reactions							
Vector #	35	36	37	38	39	40	41	42	43	44	45	1	2	3	4	5	6	7	8	9	10	11	12	13	14	15	16	17	18	19
**pi-pp**	0	0	0	0	0	0	0	0	0	0	0	-1							+1				-1							+1
**pi-cp**	0	0	0	0	0	0	0	0	0	0	0	+1												+1						
**po-pp**	0	0	0	0	0	0	0	0	0	0	0								-1										-1	
**Pst-P**	1	0	0	0	1	0	0	1	0	0	0		-1							+1										
**Pst**	0	1	1	1	0	1	1	0	1	1	1		+1							-1										
**PhoU-I**	1	1	0	0	1	1	0	1	1	0	0			-1							+1									
**PhoU-A**	0	0	1	1	0	0	1	0	0	1	1			+1							-1									
**PhoR**	1	1	1	0	1	1	1	1	1	1	0				-1	+1									+1					
**PhoR-P**	0	0	0	1	0	0	0	0	0	0	1				+1	-1									-1					
**PhoR-S**	1	1	1	1	0	0	0	1	1	1	1																			
**PhoB**	0	0	0	0	1	1	1	1	1	1	1					-1						+1				-1				
**PhoB-P**	0	0	0	0	0	0	0	0	0	0	0					+1						-1				+1				
**PhoB-S**	1	1	1	1	1	1	1	0	0	0	0																			
**PhoA-T**	1	1	1	1	1	1	1	1	1	1	1																			
**PhoA**	0	0	0	0	0	0	0	0	0	0	0						+1	-1									-1			
**PhoA-pp**	0	0	0	0	0	0	0	0	0	0	0							+1										+1		

**Term. state**				T			T				T																			

States are assigned to the experiments during which they occurred. The state vectors of experiments 1 to 8 are shown in Table 3. Difference vectors and additional decomposed reactions that appeared in more than one experiment are listed only once. States of Pst, PhoU, PhoR, and PhoB were considered as P-invariants. Phosphate, PhoR, PhoRP and the cytoplasmic PhoA protein were excluded as catalytic factors based on biological knowledge.

Each single reaction vector was then analysed whether it is applied to carry the system from one state x_i _to a subsequent state x_i+1_. If a reaction was applicable to a state x_i _which is also a terminal state, the reaction must be disabled in the terminal state, as defined by the appropriate control function (see Figure 6g). If the reaction could not be switched off, the reaction was deleted from the set of solutions. Finding appropriate control functions for all corresponding reaction vectors as described above, and excluding contradictory reactions from the set finally lead to the wiring of places and transitions and to the control of certain transitions by read arcs or inhibitory arcs.

The original phosphate regulatory network and the result of the reconstruction procedure are shown in Figure 8. The algorithm reproduced the wiring of the original Petri net. For four transitions of the original Petri net however, the algorithm found alternatives some of which in fact suggest alternative molecular mechanisms that would give rise to the same dynamic behaviour in the considered experiments while others merely make sense and can be ruled out accordingly.

The algorithm suggested that inorganic phosphate (P_i_) is transported by the PstSCAB complex into the cell (Figure 8b). The alternative mechanism that P_i _disappears in the periplasm and appears in the cytoplasm mediated by independent, non-coupled reactions both catalyzed by the PstSCAB complex would imply the existence of additional pools of inorganic phosphate different from cytoplasm and periplasm that would serve as source and sink, respectively. This alternative is ruled out as unlikely mechanism.

Alternative mechanisms of PhoU inactivation that occur by conformational coupling with the PstSCAB complex are more difficult to distinguish (Figure 8c). The algorithm suggests biochemically meaningful mechanisms, namely that inactivation of PhoU may be catalyzed by the phosphorylated form of the PstSCAB complex, or inhibited by the dephosphorylated form of PstSCAB. In principle, both mechanisms could be true but in the context of a minimal model, only one regulatory interaction, catalysis or inhibition is allowed. The third alternative suggests that inactivation of PhoU is catalyzed by periplasmic P_i_. This alternative is discarded since the two molecules reside in different spatial compartments.

A reaction where the molecular mechanism is unclear based on the results of the reconstruction algorithm concerns the dephosphorylation of PhoBP (Figure 8d). PhoBP dephosphorylation may be catalyzed by inactive PhoU or by the phosphorylated form of the PstSCAB complex or by periplasmic P_i_. While direct interaction with periplasmic P_i _again is discarded, other alternatives cannot be ruled out by argumentation. PhoBP dephosphorylation may be inhibited by active PhoU or by the dephosphorylated form of the PstSCAB complex. *In silico *phenotypes of deletion mutants did not allow to discriminate between these alternatives, suggesting to investigate these alternatives with the help of *in vitro *experiments where the biochemical activity of the individual proteins on the phosphorylated form of PhoB is analysed. Such *in vitro *data could certainly also be fed into the reconstruction algorithm.

A final ambiguity in mechanisms can simply be resolved (Figure 8e). The algorithm suggests that the cytoplasmic PhoA protein is transported into the periplasm. Alternatively, cytoplasmic PhoA would disappear into an unknown pool and appear in the periplasm from an unknown pool in independent non-coupled reactions. As a protein appearing in the periplasm must have been synthesized in the cytoplasm at some point, transport across the cytoplasmic membrane in a prokaryotic cell seems to be the right mechanism rather than exocytosis through cytoplasmic vesicles.

In summary, the algorithm found a core network with defined wiring (Figure 8a). Wiring alternatives found for four transitions can be discarded based on biological knowledge or suggested biochemically meaningful alternatives: activation of a reaction by one form of a protein or inhibition of the same reaction by its covalently modified or otherwise differently active form.

## Discussion

We have described a new algorithm for the reconstruction of extended Petri nets from time series data sets. The algorithm delivers a complete list of solutions expressed in the form of Petri nets all of which are compatible with the input data. As for the previously published method [6] there is a guarantee through mathematical proof that the list of provided minimal models is complete, in that no possible alternative Petri net is missing that might also be able to explain the data [13]. The new algorithm however provides a significant advance in delivering Petri nets with read arcs and inhibitory arcs, so-called extended Petri nets. Extended Petri nets are more powerful and straigt-forward in representing regulatory interactions in signal transduction and genetic networks. Encoding regulatory interactions including catalysis and inhibition with the help of simple place/transition Petri nets (nets without control arcs) is possible but it requires the introduction of additional places and transitions leading to an explosion of the number of generated solutions in network reconstruction through multiple alternative combinations of symmetric motifs redundant in terms of the encoded regulatory properties. By avoiding redundancy, the present algorithm allows to reconstruct networks from time series data sets that are obtained with mutants or e.g. after pharmacological intervention both of which can be regarded as structural alterations introduced into a network by targeting elements that exert regulatory control.

Using a model of the phosphate regulatory network of enterobacteria as a test case, the algorithm correctly reconstructed the Petri net which was used to generate *in silico *time series data sets for wild-type cells, deletion mutants and site-directed mutants. For four transitions of the original network the algorithm found alternatively wired transitions that would also be consistent with the data set. The alternative wiring translates into reasonable alternative molecular mechanisms which might be tested experimentally.

Attempts to reconstruct a network from a time series data set where multiple components were measured in response to a specific perturbation (stimulation) typically gives a large number of alternative networks. The implicit representation of these networks e.g. as a column of controled reactions listed for each difference vector (Figure 7a) is useful to identify those difference vectors and in turn those components of the system, the ill-resolved wiring of which greately increases the number of alternative network structures [19].

Comparative analysis of time series measured with wild-type and mutants in different genes is an efficient way to reduce the number of alternative network structures delivered by the reconstruction algorithm. When sufficient experiments with different mutants are evaluated, the algorithm may give only one or few solutions as in the phosphate regulatory network that served as a test case in the present study.

Feeding the algorithm with even more data indeed might lead to a situation where not even a single solution is found without that additional components (in the form of additional places) are introduced. The current algorithm does not support the introduction of additional components as an earlier implementation without read arcs did. This feature is to be implemented in a future version of the algorithm.

According to the original definition, Petri nets represent concurrent processes. Transitions that have the licence to fire do not necessarily fire immediately which makes the behaviour of the network nondeterministic. Reaction rates corresponding to rate constants in chemical kinetics can be introduced by assigning a probablilistic hazard function to each transition, yielding a stochastic Petri net [9]. For network reconstruction purposes, relative reaction rates can be encoded in the form of priorities to enforce in which temporal sequence competing transitions fire [7,20,21]. Considering priorities in the reconstruction process may again considerably reduce the number of alternative solutions. The present form of the algorithm does not consider priorities.

## Conclusions

The algorithm described in this work reconstructs extended Petri nets from time series data sets by finding all alternative minimal networks that are consistent with the data. It suggested reasonable alternative molecular mechanisms for certain reactions in the network that can be tested experimentally. The algorithm integrated data obtained for wild-type and for mutant cells and may, in the way shown, be useful to integrate physiological, biochemical, pharmacological, and genetic data into a consistent Petri net model. The algorithm works with discretized data or with data that are *per se *discrete like e.g. the presence or the absence of a phenotype. We consciously keep computational methods of data discretization or statistical treatment of data strictly separated from the reconstruction algorithm. With reliable, *i.e*. statistically significant and reproducible experimental data sets, the algorithm can be used to obtain certified results based on the proven property to deliver all possible minimal networks. With enough input data, one may obtain one unique network. Having data sets that give more or even many possible networks as solutions, the implicit representation of the networks can clearly show which firm conclusions can be drawn from the experimental data in terms of proven causal connections that are identical in all deliviered networks, and alternatives for the causal connection of components may be used for experimental design. The results presented in this work were obtained by manual computation. We are currently exploring the potential of Answer Set Programming (ASP) to efficiently compute large data sets to reconstruct large networks [22].

## Methods

The Petri net model of the phosphate regulatory network was drawn using the graphical editor of the Petri net tool Snoopy [17,18]. Time series data used as input for the reconstruction algorithm was obtained as follows: After setting the initial marking for an *in silico *experiment, the flow of tokens through the net was generated by selecting the firing transitions in the animation mode of Snoopy and the successive marking states of the nets were annotated manually to give the state vectors listed as columns in Tables 3 and 4. In those cases where the net entered a dead state because no transition was able to fire any more, the current state vector was flagged as a terminal state. This procedure was repeated for each of the *in silico *experiments with initial markings as shown in Table 1 and the state vectors obtained during the simulation runs again were sequentially listed in Tables 3 and 4.

Starting with the state vector matrix, the difference vector matrix was computed by substracting each state vector from its successor. Each difference vector was then split into a corresponding set consisting of all possible reaction vectors having entries which are either equal to or partially contribute to the entries of the difference vector. The complete set of reaction vectors is called reaction vector matrix.

For each reaction vector, the set of terminal states was identified at which that reaction vector is applicable. This was done by testing for each terminal state and any reaction vector, whether the entries of the sum of the two vectors is within the bounds to give a valid state vector.

For each difference vector, the list of all combinatorial possibilities to write the difference vector as a sum of vectors from its reaction vector matrix was computed.

For each combination of reaction vectors as a decomposition of a difference vector, all permutations in the sequences of the reaction vectors were generated.

For each permutation, the sequence of intermediate states was computed by starting from the first state of the difference vector and successively adding the reaction vectors in the order as they appear in that sequence. For each reaction vector in such a sequence, the information about its control function was evaluated by comparing the corresponding intermediate state vector (at which the function is 1) with the previously computed set of terminal states (at which the function is 0). If for any reaction the intermediate state was equal to a terminal state, then no control function exists and therefore the sequence was deleted. Otherwise, all logical representations with a minimal number of terms that give the required values for the control function were computed using the Quine-McCluskey Algorithm [23].

The described procedures delivered, for each difference vector, the complete list of sequences of controled reactions which are able to generate that difference vector. For each of those sequences of controled reactions, the corresponding Petri net representation was drawn in Snoopy as an alternative sub network as part of a possible solution.

All described computational steps were executed manually.

## Authors' contributions

MD, AW and WM co-developed the reported method for reconstruction of extended Petri nets. MD and AW worked out the underlying mathematics. MD reconstructed the network presented in the case study supervised by WM. WM designed the case study, wrote the manuscript and designed the figures. MD and AW helped with the preparation of the figures. AW and WM conceived of the study and participated in its design and coordination. All authors read and approved the final manuscript.

## Supplementary Material

Additional file 1 **Supplementary material to Figure 8**. The pdf contains a detailed explanation of the alternative network motifs of Figure 8 b-e. Click here for file
