# Supplementary material for: Reconstruction of extended Petri nets from time series data and its application to signal transduction and to gene regulatory networks
Source: BMC Syst Biol. 2011 Jul 15;5:113. doi: 10.1186/1752-0509-5-113 (PMC3161898; doi:10.1186/1752-0509-5-113)
Supplement: Additional file 1 — Supplementary material to Figure 8. The pdf contains a detailed explanation of the alternative network motifs of Figure 8 b-e. [file 1752-0509-5-113-S1.PDF]

Additional file 1

Supplementary material to Figure 8:

Detailed explanations of the alternative network motifs of Figure 8 b-e

Figure 8 Implicit representation of the phosphate regulatory network reconstructed and graphically displayed in extended Petri net format. a) This Petri net model of the phosphate regulatory network was used to perform the *in silico* experiments and reconstructed from the obtained data sets. b) The algorithm revealed two alternative controled reactions for difference vector $\mathbf{d}_1$. The alternative to the true mechanism of phosphate transport from the periplasm into the cytoplasm (1) suggests that inorganic phosphate disappears in the periplasm and appears in the cytoplasm, both reactions catalyzed by Pst-P (2). Since we know that inorganic phosphate is transported over the membrane, this alternative (2) is discarded. c) There are three alternative mechanisms found for $\mathbf{d}_{10}$. Inactivation of PhoU by conversion of its active form (PhoU-A) into its inactive form (PhoU-I) might be catalyzed either (1) by the phosphorylated form of the Pst protein complex (Pst-P) or (2) by inorganic phosphate in the periplasm. Since we know that PhoU is a cytoplasmic protein, alternative (2) can be discarded. Alternative (3) suggests that the inactivation of the PhoU protein is inhibited by the dephosphorylated form (Pst) of the Pst protein complex rather than catalyzed by the phosphorylated form (Pst-P) as suggested by alternative (1). Both mechanisms ((1) and (3)) seem reasonable from the biochemical point of view and cannot be discriminated based on the data set used. d) There are five alternative controled reactions that may account for difference vector $\mathbf{d}_{11}$. Dephosphorylation of PhoB-P may either be catalyzed (1) by inorganic phosphate in the periplasm, (2) by the phosphorylated form of

the Pst protein complex or (3) by inactive PhoU protein. Alternatively, dephosphorylation of PhoB-P may be inhibited (4) by the dephosphorylated form of the Pst protein complex or (5) by the PhoU protein in its active form. Alternative (1) is discarded as PhoB is a cytoplasmic protein. To experimentally discriminate the other alternatives, the reconstruction result suggests to perform *in vitro* studies to determine whether the PhoU protein is a PhoB-P phosphatase or whether the dephosphorylation reaction depends on physical interaction with components of the Pst protein complex. e) Finally, there are two alternative mechanisms to account for difference vector $\mathbf{d}_7$. (1) The PhoA protein synthesized in the cytoplasm could be transported into the periplasm which is the true mechanism. (2) The alternative suggested by the algorithm is that the PhoA protein disappears from the cytoplasm (e.g. by being degraded or secreted into a fictive spatial compartment) and somehow appears in the periplasm. Alternative (2) is discarded as we know that periplasmic proteins are directly transported from the cytoplasm into the periplasm. Note that alternatives (2) in a) and e) might also be excluded solely based on the minimality criterion.
